# Association between multimorbidity trajectories and incident disability among mid to older age adults: China Health and Retirement Longitudinal Study

**DOI:** 10.1186/s12877-022-03421-9

**Published:** 2022-09-12

**Authors:** Zaixing Shi, Zeyun Zhang, Kanglin Shi, Bohan Yu, Zhongquan Jiang, Li Yang, Jianlin Lin, Ya Fang

**Affiliations:** 1 School of Public Health, Xiamen University, Xiamen, 361102 China; 2 State Key Laboratory of Molecular Vaccine and Molecular Diagnostics, School of Public Health, Xiamen University, Xiamen, 361102 China; 3 Key Laboratory of Health Technology Assessment of Fujian Province, School of Public Health, Xiamen University, Xiamen, 361102 China

**Keywords:** Multimorbidity, Disability, Group-based multi-trajectory modeling, Generalized estimating equation

## Abstract

**Background:**

Although multimorbidity is a risk factor for disability, the relationship between the accumulative patterns of multimorbidity and disability remains poorly understood. The objective of this study was to identify the latent groups of multimorbidity trajectories among mid to older age adults and to examine their associations with incident disability.

**Methods:**

We included 5,548 participants aged ≥ 45 years who participated in the China Health and Retirement Longitudinal Study from 2011 to 2018 and had no multimorbidity (≥ 2 chronic conditions) at baseline. The group-based multi-trajectory modeling was used to identify distinct trajectory groups of multimorbidity based on the latent dimensions underlying 13 chronic conditions. The association between multimorbidity trajectories and incident disability was analyzed using the generalized estimating equation model adjusting for potential confounders.

**Results:**

Of the 5,548 participants included in the current analysis, 2,407 (43.39%) developed multimorbidity during the follow-up. Among participants with new-onset multimorbidity, four trajectory groups were identified according to the combination of newly diagnosed diseases: “Cardiometabolic” (*N* = 821, 34.11%), “Digestive-arthritic” (*N* = 753, 31.28%), “Cardiometabolic/Brain” (*N* = 618, 25.68%), and “Respiratory” (*N* = 215, 8.93%). Compared to participants who did not develop multimorbidity, the risk of incident disability was most significantly increased in the “Cardiometabolic/Brain” trajectory group (OR = 2.05, 95% CI: 1.55–2.70), followed by the “Cardiometabolic” (OR = 1.96, 95% CI: 1.52 –2.53) and “Digestive-arthritic” (OR = 1.70, 95% CI: 1.31–2.20) trajectory groups.

**Conclusions:**

The growing burden of multimorbidity, especially the comorbid of cardiometabolic and brain diseases, may be associated with a significantly increased risk of disability for mid to older age adults. These findings improve our understanding of multimorbidity patterns that affect the independence of living and inform the development of strategies for the primary prevention of disability.

**Supplementary Information:**

The online version contains supplementary material available at 10.1186/s12877-022-03421-9.

## Background

Older adults living with disability and impaired physical function may have a significantly increased risk of mortality and worse quality of life [1–4]. Over 45% of older adults aged 60 years and above have difficulty performing daily activities [5]. A growing body of literature suggests a strong association between chronic diseases and loss of physical functioning. Prior studies have confirmed that the level of disability increases with a higher number of chronic diseases [6, 7]. With advances in medical care and longer life expectancy, a growing percentage of adults are living with multiple chronic diseases [8]. The prevalence of multimorbidity, defined as the co-existence of two or more medical conditions within a person [9], is estimated to be 34 to 61% in older adults [10], which is associated with a higher risk of disability, [11–15] poor quality of life, [16, 17] and mortality [18]. Moreover, former studies have demonstrated a trend toward the earlier onset of multimorbidity [19]. Multimorbidity has become common in mid-life in many countries [20, 21]. For instance, a Canadian study showed that the prevalence of multimorbidity was 30% among adults aged 45 to 49 years, 34% among 50 to 54 years, and 45% among 55 to 59 years [20]. It is important to understand how multimorbidity contributes to the development of disability for mid to older age adults.

Most research to date has analyzed multimorbidity as a binary variable or the count of chronic conditions, which may be insufficient to reflect the heterogeneity of chronic diseases comprising multimorbidity. Recent studies suggest that persons affected by specific combinations of chronic conditions may have a particularly high risk of disability [22, 23]. For example, Marengoni and colleagues identified six unique multimorbidity patterns: psychiatric, musculoskeletal/respiratory/gastrointestinal, sensory impairment/cancer, metabolic/sleep disorders, cardiovascular/anemia/dementia, and an unspecific pattern. Compared to older adults with the unspecific pattern, those with the cardiovascular/anemia/dementia, musculoskeletal/respiratory/gastrointestinal, and sensory impairment/cancer patterns had an increased risk of impairment in activities of daily living (ADL) and instrumental activities of daily living (IADL) [24]. However, previous studies of multimorbidity and disability have several limitations. First, few studies have investigated the prospective relationship between multimorbidity patterns and disability [22]. Second, little is known about the developmental trajectories of multimorbidity and their impact on disability. Although some studies have started to explore the trajectories of multimorbidity in older adults, most relied on the binary multimorbidity status or the number of diseases. The accumulation patterns of multiple chronic diseases remain largely unknown. Finally, chronic diseases often emerge before the age of 60 after long-term exposure to risk factors, yet few studies have evaluated the trajectories of multimorbidity starting from middle age.

Based on a large and nationally representative sample of mid to older age adults from the China Health and Retirement Longitudinal Study, this study aimed to (1) identify the multimorbidity trajectory groups among mid to older age adults with new-onset multimorbidity, and (2) explore the association between multimorbidity trajectories and incident disability.

## Methods

### Data source

Data were obtained from the China Health and Retirement Longitudinal Study (CHARLS), a prospective cohort study of adults aged 45 years and above and their partners in China. The study used a multistage probability sampling strategy to select participants. Participants were enrolled in 2011 and were followed up in 2013, 2015, and 2018. Detailed study design of the CHARLS is available elsewhere [25].

**Fig. 1:**
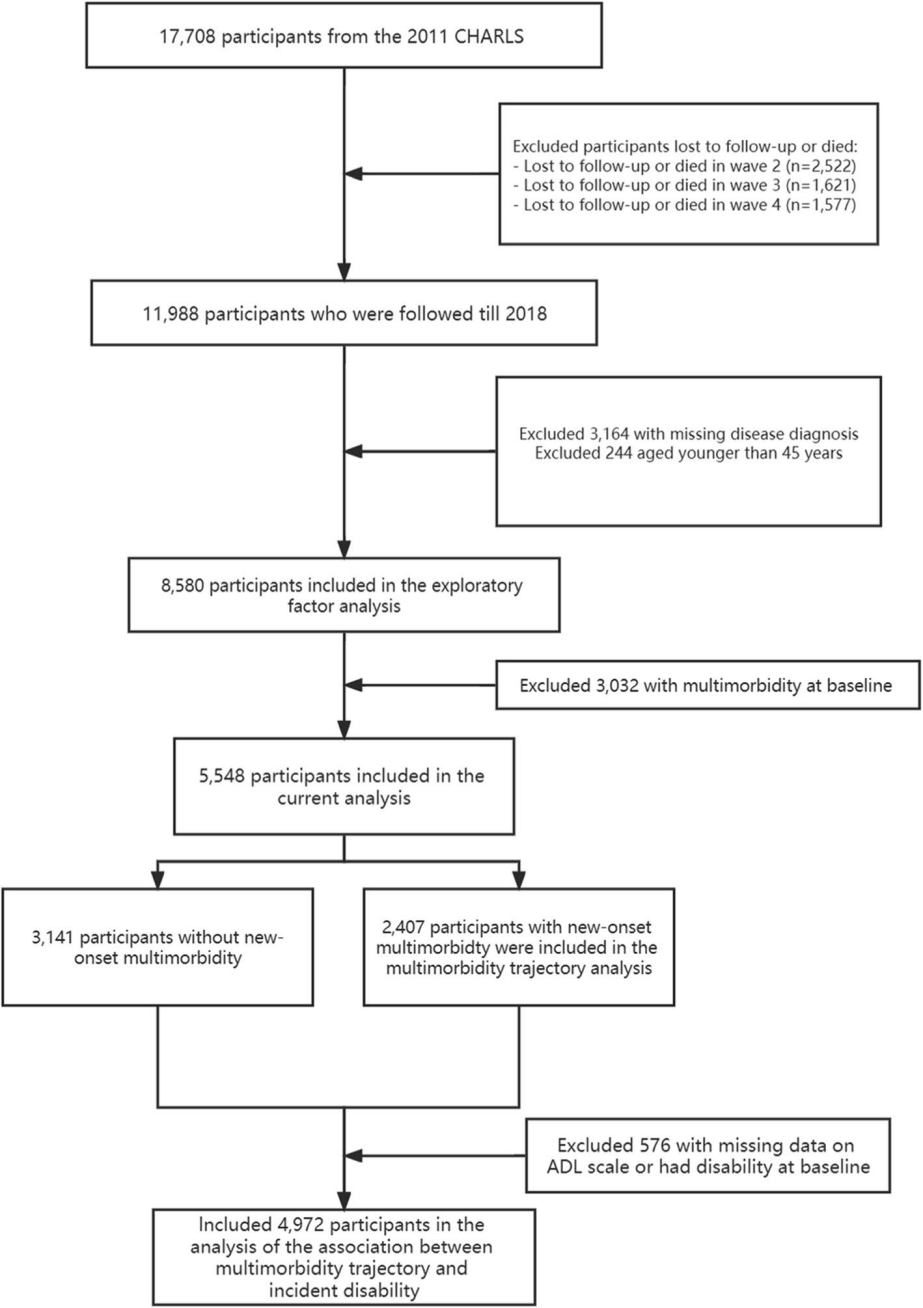
Flow chart of sample selection for the current analysis

A total of 17,708 individuals completed the baseline survey. Of these, 15,186 were surveyed at wave 2 (2013), 13,565 at wave 3 (2015), and 11,988 at wave 4 (2018). All participants with one or more missing values in chronic conditions (*N* = 3,164) were excluded from the data analysis. In addition, all participants who were under the age of 45 years (*N* = 244) or had multimorbidity (*N* = 3,032) at baseline were also excluded. Thus, we included 5,548 participants in the current analysis. Further, in analyzing the association between multimorbidity trajectories and incident ADL disability, additional 576 participants were excluded for the following reasons: having missing values in the baseline ADL scale variable, or having ADL disability at baseline. Detailed procedure of sample selection is shown in Fig. 1. The biomedical ethics committee of Peking University approved the CHARLS and written informed consent was obtained from all participants.

### Measures

#### Chronic conditions and multimorbidity

We ascertained the history of 14 chronic diseases by asking “Have you been diagnosed with the following conditions by a doctor”: hypertension, dyslipidemia, diabetes, cancer, chronic lung diseases, liver disease, heart disease, stroke, kidney disease, stomach disease, emotional problems, memory-related disease, arthritis, and asthma. These diseases are the leading causes of death in Chinese and are generally irreversible [26]. The self-reported data on chronic diseases are in high agreement with that based on medical records [27]. All diseases and conditions were defined as a binary variable (1 = present, 0 = absent). Multimorbidity was defined as having two or more of any aforementioned diseases. Participants who were qualified as having multimorbidity during follow-up would be defined as having a new onset of multimorbidity, with all participants free of multimorbidity at baseline.

#### Physical function assessment

We used the Chinese version of the Katz Activities of Daily Living (ADL) scale to measure disability [28]. This scale assessed perceived difficulties in the six ADLs, including dressing, bathing, eating, getting into and out of bed, toileting, and controlling urination and defecation. Following previous studies [29], participants were classified as having ADL disability if they reported having any degree of difficulties in performing at least one ADL. Otherwise, they were considered as having no ADL disability. Participants who had no disability at baseline but were qualified as having ADL disability at any wave of follow-up were considered as developing an incident ADL disability.

#### Covariates

We included the following baseline variables as covariates: age (45–59, 60 + years), sex (male, female), self-rated health (poor, fair, good) [30], education (no formal education, primary school, middle school or above), body mass index (BMI) (underweight: < 18.5 kg/m^2^, normal: 18.5–22.9 kg/m^2^, overweight: 23.0–27.4 kg/m^2^, obesity: ≥ 27.5 kg/m^2^), [31] occupation (agricultural, non-agricultural, unemployed/retired), ever smoking (no, yes), alcohol drinking (no, yes), sleep duration (< 7 h/day, 7–8 h/day, > 8 h/day), marital status (married/cohabitation, single: divorced, separated, widowed, or never married), annual household expenditure (≤ 2800 yuan, 2801–4846 yuan, 4847–8325 yuan, > 8325 yuan), social participation (whether the respondent participated in any social activities), location of residence (rural, urban) and public health insurance status (no, yes).

### Statistical analyses

The multimorbidity trajectory groups were derived according to the longitudinal data of chronic conditions. But due to the high dimensionality of the longitudinal data of chronic conditions, we cannot directly derive the trajectory groups. To address this challenge, we first used exploratory factor analysis (EFA) to construct multimorbidity patterns to reduce the dimensionality of the 14 chronic conditions. We then calculated the corresponding factor scores of the multimorbidity patterns and used the factor scores as the outcome variables in deriving trajectory of the multimorbidity using the group-based multi-trajectory modeling (GBMTM).

We used EFA to determine the latent multimorbidity patterns underlying the 14 chronic conditions in the baseline sample. The patterns were determined based on their interpretability. We used the weighted least squares means and variance (WLSMV) estimator to estimate the EFA model [32]. We evaluate the goodness of fit by the comparative fit index (CFI), standardized root mean square residual (SRMR), root mean squared error of approximation (RMSEA), and tucker and lewis index (TLI) [33–35]. For better interpretations, an oblique rotation of factor loading matrices was performed, with each resulting factor loading representing the strength of association between each condition and the latent multimorbidity patterns. A factor loading of ≥ 0.40 indicates a strong association. The multimorbidity patterns were named according to the conditions that were most strongly associated with them. After identifying the latent multimorbidity patterns at baseline, we estimated the factor scores for each pattern over the follow-up using factor loadings of the multimorbidity patterns at baseline. The multimorbidity pattern score ranged between -0.31 and 2.68, with a higher score suggesting a greater number of conditions belonging to a specific multimorbidity pattern. We excluded conditions with a prevalence of < 1.0% in the baseline sample to achieve better robustness [36].

Based on the longitudinal factor scores of multimorbidity patterns, we identified subgroups of participants with similar joint trajectories of the multidimensional scores using the GBMTM. In this model, the outcome variables were the factor scores of each multimorbidity pattern over the follow-up, and these variables were modeled using the censored normal distribution by setting the lowest score (-0.31) as the censored minimum and the highest score (2.68) as the censored maximum. Based on previous research on multimorbidity trajectories in Korean older adults [37], we hypothesized that there would be 2–6 distinct trajectories of multimorbidity. Model fitting proceeded iteratively by comparing models with a varying number of groups (2–6 groups) and shapes of trajectories (linear, quadratic, and cubic). Model selection was based on the Bayesian information criterion (BIC) and Akaike’s information criterion (AIC) value, in which the model with the lowest BIC and AIC value was preferred. In addition, an ideal model is one in which the proportion assigned to each trajectory group (based on the maximum posterior probability rule) is greater than 5%; the average posterior probability of group membership is at least 0.7. Finally, the final models should have sufficient clinical relevance and interpretability [38].

We used the generalized estimating equation (GEE) model with an independent correlation matrix to evaluate the association between multimorbidity trajectories and incident disability. The independent variable was multimorbidity trajectory group, with participants without multimorbidity as the referent group. The dependent variable was disability status (yes, no) which was modeled with a logit link function. All variables included in this study were repeatedly measured in 2011, 2013, 2015, and 2018. Three models were fitted consecutively: model 1 was adjusted for time, model 2 was additionally adjusted for sociodemographic characteristics (age, gender, marital status, living area, occupation, annual household expenditure, social participation, and public health insurance status), and model 3 was further adjusted for health-related characteristics (self-rated health, BMI, smoking, alcohol drinking, and sleep duration). We reported the odds ratios (ORs) with 95% confidence intervals (CIs) of the association between multimorbidity trajectory groups and disability. We tested the interaction between multimorbidity trajectory groups and time in the GEE model, but the interaction was not statistically significant, therefore the interaction term was not included in the models.

The EFA analyses were performed with Mplus version 8.0, the GBMTM was conducted using the “Traj” program in Stata version 16.0 (StataCorp, College Station, TX) [39], and the GEE model was fitted using R 4.1.2. A two-sided *P* value < 0.05 was considered statistically significant.

### Sensitivity analysis

We conducted a set of sensitivity analyses: First, we performed three EFA using the cross-sectional data from 2013, 2015, and 2018 waves of CHARLS to assess the robustness of the EFA, where we included the same chronic conditions as those at baseline to make the results comparable with those of the main analysis. Second, to evaluate the influence of incomplete data on the trajectory analysis, we conducted sensitivity analysis by restricted participants with complete data for at least 3 waves (*N* = 5,888). Third, to evaluate the effects of the excluded sample, the characteristics from samples with and without participants who were excluded during sample selection were compared and fitted the GEE model regarding the association of multimorbidity and incident disability among those excluded from the current analysis. Fourth, we conducted an analysis that only excluded participants with multimorbidity at baseline (*N* = 6,630) to assess the impact of the excluded sample on the study results. Finally, we divided participants without multimorbidity into no morbidity and single morbidity groups to examine the impact of single morbidity on the association between multimorbidity trajectories and incident disability using the GEE model.

## Results

### Baseline characteristics

Of the 5,548 participants included in the current analysis, 2,407 (43.39%) developed multimorbidity during the follow-up. Detailed sample characteristics, as well as the differences between participants with and without new-onset multimorbidity, are presented in Supplemental Table 1. Briefly, participants who developed multimorbidity tended to be female, older, overweight or obese, unemployed or retired, and report poorer self-rated health, disability, and shorter sleep duration at baseline compared to those without multimorbidity (all *P* < 0.05). Compared to those included in the current analyses, excluded participants were more likely to be female, older, overweight or obese, unemployed or retired, single, and report no formal education, poorer self-rated health, disability, and shorter sleep duration (Supplemental Table 2).

### Exploratory factor analysis of latent multimorbidity patterns

**Fig. 2:**
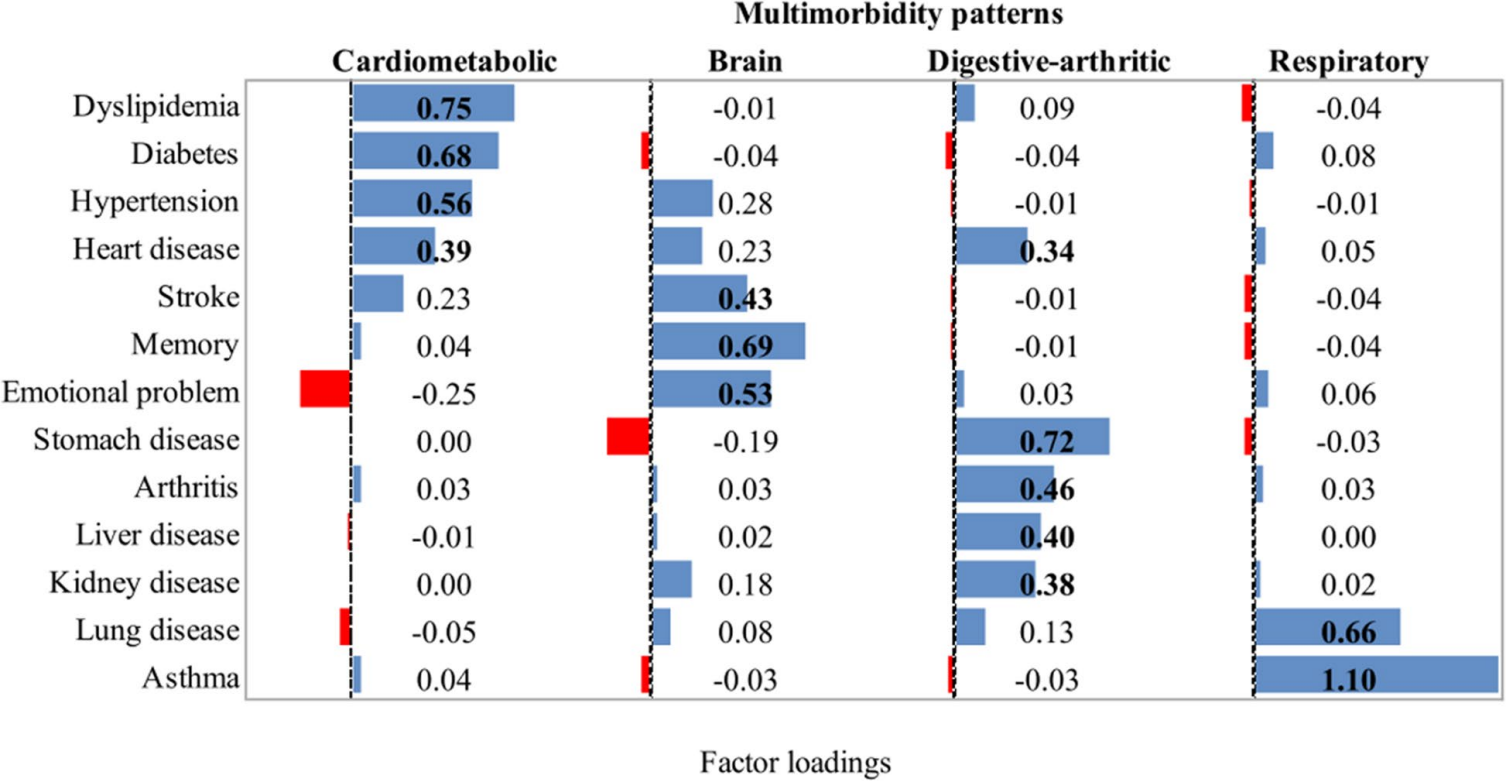
Factor loadings of the 4 multimorbidity patterns identified by the exploratory factor analysis

The exploratory factor analysis included 13 chronic diseases with a prevalence of > 1% in the current sample (Fig. 2). We identified four latent multimorbidity patterns: 1) a “Cardiometabolic” pattern characterized by high correlations with heart diseases, hypertension, diabetes, and dyslipidemia; 2) a “Brain” pattern characterized by stroke, memory-related disease, and emotional problems; 3) a “Digestive-arthritic” pattern which was strongly correlated with kidney disease, liver disease, digestive disease, and arthritis; and 4) a “Respiratory” pattern which was correlated with chronic lung diseases and asthma. We identified similar patterns using the cross-sectional data from 2013, 2015, and 2018 waves of CHARLS (Supplemental Figures 1–3), suggesting the robustness of the EFA.

### Group-based multi-trajectory modeling of multimorbidity trajectories

**Fig. 3:**
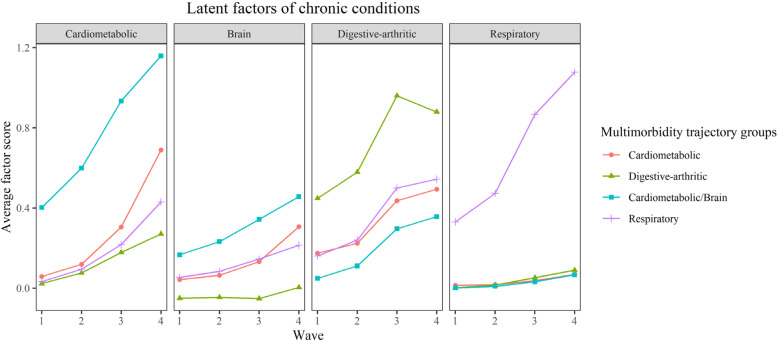
Average factor scores for each multimorbidity trajectory groups in mid to older age adults

Among mid to older age adults who developed multimorbidity, four distinct groups of multimorbidity trajectories were identified based on the joint trajectories of the four multimorbidity pattern scores (Fig. 3). The resulting trajectory groups were named according to the patterns with the most prominent increasing trend, indicating that the trajectory group was dominated by the disease growth of these multimorbidity patterns in the process of no multimorbidity to multimorbidity. Specifically, the first group showed the most prominent increasing trend in the “Cardiometabolic” multimorbidity pattern score, and therefore named “Cardiometabolic” trajectory (*N* = 821, 34.11%). Similarly, the second group was named “Digestive-arthritic” trajectory (*N* = 753, 31.28%), the third group was named “Cardiometabolic/Brain” trajectory (*N* = 618, 25.68%), and the fourth group was named “Respiratory” trajectory (*N* = 215, 8.93%). In a sensitivity analysis that restricted to participants with complete data for at least 3 waves, the multimorbidity trajectories were similar to those from the main analyses (Supplemental Fig. 4).

### Baseline characteristics associated with multimorbidity trajectories

Table 1 presents the baseline characteristics by multimorbidity trajectory groups. There were significant differences in sex, age, self-rated health, education, BMI, occupation, disability, cigarette smoking, sleep duration, and location of residence across multimorbidity trajectories. Of particular note, participants with the “Digestive-arthritic” and the “Respiratory” trajectories were much more likely to report poor self-rated health, and participants with the “Cardiometabolic/Brain” and the “Cardiometabolic” trajectories were much more likely to have a BMI ≥ 27.5 kg/m^2^ (all *P* < 0.001).

**Table 1:** Baseline sample characteristics according to multimorbidity trajectory groups

Characteristic	No multimorbidity (*N* = 3141)	Cardio-metabolic (*N* = 821)	Digestive-arthritic (*N* = 753)	Cardio-metabolic/Brain (*N* = 618)	Respiratory(*N* = 215)	*P* Value
**Sex, n (%)**^a^						< .001
Male	1629 (51.9)	416 (50.7)	328 (43.6)	298 (48.2)	132 (61.4)	
Female	1512 (48.1)	405 (49.3)	425 (56.4)	320 (51.8)	83 (38.6)	
**Age(years), mean ± SD**^b^	56.4 ± 8.7	57.8 ± 8.5	56.5 ± 8.0	58.6 ± 8.9	59.2 ± 9.1	< .001
**Self-rated health, n (%)**^a^						< .001
Poor	218 (9.7)	86 (14.4)	124 (23.2)	70 (15.4)	42 (26.1)	
Fair	996 (44.3)	328 (54.8)	295 (55.1)	232 (51.0)	75 (46.6)	
Good	1032 (46.0)	184 (30.8)	116 (21.7)	153 (33.6)	44 (27.3)	
**Education, n (%)**^**a**^						< .001
No formal education	1254 (39.9)	383 (46.7)	364 (48.3)	248 (40.1)	106 (49.3)	
Primary school	687 (21.9)	183 (22.3)	153 (20.3)	140 (22.7)	47 (21.9)	
Middle school or above	1200 (38.2)	255 (31.0)	236 (31.4)	230 (37.2)	62 (28.8)	
**BMI(kg/m**^**2**^**), n (%)**^a^						< .001
< 18.5	152 (5.8)	36 (5.3)	51 (8.0)	10 (2.0)	19 (11.2)	
18.5–22.9	1272 (48.7)	284 (42.1)	323 (50.9)	161 (31.5)	85 (50.3)	
23.0–27.4	938 (35.8)	276 (40.9)	222 (35.1)	222 (43.4)	50 (29.6)	
≥ 27.5	255 (9.7)	79 (11.7)	38 (6.0)	118 (23.1)	15 (8.9)	
**Occupation, n (%)**^a^						< .001
Agricultural work	1657 (53.0)	445 (54.2)	449 (59.9)	276 (45.0)	118 (54.9)	
Non-agricultural work	870 (27.8)	193 (23.5)	155 (20.6)	141 (23.0)	42 (19.5)	
Unemployed/retired	601 (19.2)	183 (22.3)	146 (19.5)	197 (32.0)	55 (25.6)	
**ADL disability, n (%)**^a^						< .001
No	2905 (93.6)	705 (86.9)	633 (84.6)	543 (88.7)	186 (87.7)	
Yes	200 (6.4)	106 (13.1)	115 (15.4)	69 (11.3)	26 (12.3)	
**Smoking, n (%)**^a^						< .001
No	1846 (58.8)	472 (57.5)	483 (64.1)	375 (60.7)	100 (46.5)	
Yes	1294 (41.2)	349 (42.5)	270 (35.9)	243 (39.3)	115 (53.5)	
**Alcohol drinking, n (%)**^a^						0.894
No	1862 (59.3)	482 (58.8)	459 (61.0)	371 (60.1)	130 (60.5)	
Yes	1276 (40.7)	338 (41.2)	293 (39.0)	246 (39.9)	85 (39.5)	
**Sleep duration(h/day), n (%)**^a^						< .001
< 7	1242 (42.2)	381 (50.1)	389 (54.9)	258 (44.1)	88 (44.9)	
7-8	1443 (48.9)	310 (40.7)	265 (37.3)	266 (45.5)	87 (44.4)	
> 8	263 (8.9)	70 (9.2)	55 (7.8)	61 (10.4)	21 (10.7)	
**Marital status, n (%)**^a^						0.570
Married/cohabitation	2871 (91.4)	738 (89.9)	688 (91.4)	556 (90.0)	194 (90.2)	
Single	270 (8.6)	83 (10.1)	65 (8.6)	62 (10.0)	21 (9.8)	
**Household expenditure (yuan), n (%)**^a^						0.172
≤ 2800	712 (26.7)	212 (29.6)	177 (27.3)	134 (25.2)	67 (36.1)	
2801–4846	726 (27.2)	174 (24.2)	176 (27.2)	134 (25.2)	41 (22.0)	
4847–8325	647 (24.3)	182 (25.4)	162 (25.0)	141 (26.5)	35 (18.8)	
> 8325	582 (21.8)	149 (20.8)	133 (20.5)	123 (23.1)	43 (23.1)	
**Social engagement, n (%)**^a^						0.828
No	1571 (53.0)	412 (53.9)	396 (55.2)	307 (52.3)	108 (53.5)	
Yes	1391 (47.0)	352 (46.1)	321 (44.8)	280 (47.7)	94 (46.5)	
**Location of residence, n (%)**^a^						0.013
Rural	2082 (66.3)	534 (65.0)	511 (67.9)	368 (59.5)	138 (64.2)	
Urban	1059 (33.7)	287 (35.0)	242 (32.1)	250 (40.5)	77 (35.8)	
**Health insurance, n (%)**^**a**^						0.320
No	229 (7.3)	65 (7.9)	48 (6.4)	34 (5.5)	12 (5.6)	
Yes	2905 (92.7)	753 (92.1)	704 (93.6)	582 (94.5)	201 (94.4)	

^a^Pearson chi-square tests for categorical variables

^b^One-way analysis of variance tests for continuous variables

### Association between multimorbidity trajectories and incident disability

Table 2 summarizes the association between multimorbidity trajectory groups and incident disability using the GEE model. In the fully adjusted model, the “Cardiometabolic/Brain” (OR = 2.05, 95% CI: 1.55–2.70) trajectory group had the highest increased risk of disability, followed by the “Cardiometabolic” (OR = 1.96, 95% CI: 1.52 -2.53) and “Digestive-arthritic” (OR = 1.70, 95% CI: 1.31–2.20) trajectory groups, but no significant associations were observed with the “Respiratory” trajectory group.

**Table 2:** Association of multimorbidity trajectories and incident disability based on the GEE model

	**Model 1**^a^	**Model 2**^b^	**Model 3**^c^
**Multimorbidity trajectory**	**OR (95% CI)**	**OR (95% CI)**	**OR (95% CI)**
**No multimorbidity (referent)**	1.00	1.00	1.00
**New-onset multimorbidity**	2.20***(1.92—2.53)	2.29***(1.93—2.71)	1.85***(1.53—2.23)
**Multimorbidity trajectory groups (ref = no multimorbidity)**			
Cardiometabolic	2.01***(1.66—2.43)	2.30***(1.83—2.90)	1.96***(1.52—2.53)
Digestive-arthritic	2.12***(1.75—2.58)	2.29***(1.81—2.90)	1.70***(1.31—2.20)
Cardiometabolic/Brain	2.61***(2.14—3.19)	2.40***(1.86—3.09)	2.05***(1.55—2.70)
Respiratory	2.07***(1.54—2.77)	1.96***(1.34—2.87)	1.44(0.93—2.23)

*Abbreviations*: *OR* Odds ratio, *CI* Confidence interval

^a^Adjusted for time

^b^Adjusted for time and sociodemographic characteristics variables, including age, gender, marital status, living area, occupation, annual household expenditure, social participation, and public health insurance status

^c^Adjusted for time, sociodemographic characteristics variables, and health-related characteristics (self-rated health, BMI, smoking, alcohol drinking, and sleep duration)

^***^ *p* < .001, ** *p* < .01, * *p* < .05

Among excluded participants, those with multimorbidity had a significantly increased risk of disability compared to those without multimorbidity (OR = 1.64, 95% CI: 1.41–1.90). (Supplemental Table 3) In the analysis that only excluded participants with multimorbidity at baseline, the multimorbidity trajectories and their impact on incident disability were similar to those from the main analyses. (Supplemental Figure 5 and Table 4) In a sensitivity analysis evaluating the impact of single morbidity on the association between multimorbidity trajectories and incident disability, compared with participants without any morbidity, participants with single morbidity had a significantly increased risk of disability (OR = 1.33, 95% CI: 1.02 -1.73), and the multimorbidity trajectory groups were still significantly associated with a higher risk of disability. (Supplemental Table 5).

## Discussion

Based on a large, nationally representative sample of Chinese adults, we examined the joint developmental trajectories of multimorbidity among mid to older age adults. Four groups of multimorbidity trajectories were identified, which were characterized by the most prominent increasing trend in the factor scores of the “Cardiometabolic,” “Digestive-arthritic,” “Cardiometabolic/Brain,” and “Respiratory” multimorbidity patterns, respectively. For multimorbidity trajectory groups, compared to those who did not develop multimorbidity, except for the “Respiratory” trajectory group, the “Cardiometabolic,” “Digestive-arthritic,” and the “Cardiometabolic/Brain” trajectory groups had a significantly higher risk of disability. Our findings highlight the importance of the prevention and management of chronic diseases, especially cardiometabolic and brain diseases, in reducing disability in mid to older age adults.

Our findings confirm the complexity and heterogeneity of multimorbidity and its impact on functional health, suggesting the need to look beyond the count of chronic conditions to assess its effect on older adults’ health [40]. Our study contributes to the growing literature on the latent clustering of chronic conditions. For instance, in another study based on the CHARLS data, Yao et al. identified four multimorbidity patterns using factor analysis, including the respiratory, hepatic-renal-skeletal, cardio-metabolic, and arthritic-digestive-visual patterns. [36] Jackson et al. identified three multimorbidity patterns by factor analysis, including the neurological/mental health, cardiovascular, and musculoskeletal/somatic patterns, and found that older adults with the cardiovascular pattern showed the greatest decline in ADL over 6 years of follow-up [41]. However, few studies have examined the longitudinal change in disease clustering, which may be more efficient than studies of single disease. Our study fills in the gap by modeling the joint trajectories of multimorbidity pattern scores underlying chronic disease, which offers new insights into the dynamic accumulation process of multimorbidity.

Our findings suggest that the growing burden of cardiometabolic disease contributed most significantly to the risk of disability. This is consistent with previous studies showing a positive association of metabolic multimorbidity with a higher risk of disability [42], cardiovascular outcomes, and mortality [43]. Similar to our findings, a recent study based on the CHARLS suggested that the older adults who exhibited the “cardiometabolic” and the “stomach/arthritis” patterns of multimorbidity were at greater risk of function decline when developing a new chronic disease. However, this study did not examine the risk associated with specific types of newly-onset diseases [44]. In addition, our findings highlight a significantly increased risk of disability in adults with an increasing burden of arthritic, digestive, and brain diseases. Similar to these findings, a recent analysis based on the Health and Retirement Study suggested that a multimorbidity characterized by arthritis, hypertension, and depression may be associated with the highest risk of disabilities [12]. The accumulation of chronic diseases could be a result of aging-related physiological processes such as chronic inflammation, which may be the root causes leading to the disability [45]. It has been documented that the levels of inflammatory markers were different by multimorbidity patterns, with the highest level of inflammation in the cardiovascular pattern [46]. Other shared common etiologic mechanisms underlying the cardiometabolic, arthritic, digestive, and brain diseases may also explain the increased risk of disability, which warrants future research. The results of this study highlight the importance of developing and delivering interventions to manage multimorbidity to reduce the risk of ADL disability among mid to older age adults. Furthermore, since most chronic conditions could be due to unhealthy behaviors, such as smoking and lack of physical activity, more efforts are needed to promote healthy living among mid to older age adults.

To our knowledge, this is the first study to identify the latent developmental trajectory of multiple chronic diseases by leveraging the dimension-reducing capability of exploratory factor analysis. In addition, the current study estimated multimorbidity trajectory in mid to older age adults using a novel multi-trajectory approach. The GBMTM method accounted for the co-development of the multimorbidity pattern, which provides a greater understanding of the complexity of multimorbidity accumulation. Our findings reveal clinically meaningful patterns of multimorbidity development, which have important implications for the primary prevention of disability.

This analysis has several limitations. First, our measurement of multimorbidity was limited to 14 chronic conditions that were self-reported by study participants, which may not be reliable or comprehensive. Findings could be different if a wide scope of chronic conditions were considered along with detailed parameters of the conditions (e.g. severity or duration). Second, some trajectory groups were relatively small, which may lead to limited power. Third, functional assessments in the CHARLS study were self-reported, which may have led to an inaccurate estimate of the true prevalence of disability. Future studies should evaluate disability using more objective measurements, such as walking speed and the grip strength test. Finally, because this study selected participants who participated in all four waves of CHARLS, the results are not free from selection bias and influences of attrition. Compared to those excluded from the current analyses, included participants were healthier. Therefore, findings should be interpreted with caution with full consideration of sample characteristics and the potential for selection bias. However, in the sensitivity analysis that only excluded participants with multimorbidity at baseline, the results were similar to those from the main analyses, suggesting that our findings are robust.

## Conclusions

There was great heterogeneity in the development of multimorbidity among mid to older age adults in China. For mid to older age adults, multimorbidity trajectory groups are characterized by high increases in the “Cardiometabolic,” “Digestive-arthritic,” and “Cardiometabolic/Brain” multimorbidity patterns were associated with a significantly increased risk of disability. Findings from this study highlight the importance of developing and providing interventions for managing and preventing multimorbidity to reduce disability among mid to older age adults.

## Supplementary Information

**Additional file 1.**

## Data Availability

The analytical dataset used in this study is a publicly available dataset released by the CHARLS. Information about the data source and available data are found at https://charls.charlsdata.com/pages/data/111/zh-cn.html. Researchers can obtain these data after submitting a data use agreement to the CHARLS team.

## Abbreviations

CHARLS: China Health and Retirement Longitudinal Study
ADL: Activities of daily living
IADL: Instrumental activities of daily living
BIC: Bayesian information criterion
AIC: Akaike’s information criterion
EFA: Exploratory factor analysis
GBMTM: Group-based multi-trajectory modeling
WLSMV: Weighted least squares means and variance
CFI: Comparative fit index
TLI: Tucker and lewis index
RMSEA: Root mean squared error of approximation
SRMR: Standardized root mean square residual
GEE: Generalized estimating equation
OR: Odds ratio
CI: Confidence interval
