# Supplementary material for: Association between multimorbidity trajectories and incident disability among mid to older age adults: China Health and Retirement Longitudinal Study
Source: BMC Geriatr. 2022 Sep 12;22:741. doi: 10.1186/s12877-022-03421-9 (PMC9469590; doi:10.1186/s12877-022-03421-9)
Supplement: Supplementary file 1 — Additional file 1. [file 12877_2022_3421_MOESM1_ESM.docx]

Association between multimorbidity trajectories and incident disability among mid to older age adults: China Health and Retirement Longitudinal Study

Zaixing Shi^a,b,c†^, Zeyun Zhang^a†^, Kanglin Shi^a^, Bohan Yu^a^, Zhongquan Jiang^a^, Li Yang^a^, Jianlin Lin^a^, Ya Fang^a,b,c^

Author Affiliations:

1. School of Public Health, Xiamen University, China;
2. State Key Laboratory of Molecular Vaccine and Molecular Diagnostics, School of Public Health, Xiamen University, China;
3. Key Laboratory of Health Technology Assessment of Fujian Province, School of Public Health, Xiamen University, China.

† These authors contributed equally to this work.

Corresponding Author:

Ya Fang, MD, PhD,

School of Public Health, Xiamen University, Xiang’an South Road, Xiamen 361102, China; Email: fangya@xmu.edu.cn, Tel.: +86-592-2880636

SUPPLEMENTAL FILES

Supplemental Table 1. Baseline characteristic of the analytical sample from the CHARLS study

Supplemental Table 2. Comparison of baseline characteristics between participants included (n = 5,548) and excluded (n =12,160)

Supplemental Table 3. Association of multimorbidity and incident disability based on the GEE model among participants excluded from the current analysis

Supplemental Table 4. Association of multimorbidity trajectories and incident disability in the analysis that only excluded participants with multimorbidity at baseline

Supplemental Table 5. The impact of single morbidity on the association between multimorbidity trajectories and incident disability

Supplemental Table 6. The goodness of fit for the EFA models

Supplemental Table 7. Model fitting statistics for participants with new onset of multimorbidity

Supplemental Table 8. The final four-group trajectory model of multimorbidity pattern for participants with new onset of multimorbidity

Supplemental Figure 1 . Factor loadings of the 4 multimorbidity patterns for each condition in CHARLS 2013

Supplemental Figure 2. Factor loadings of the 4 multimorbidity patterns for each condition in CHARLS 2015

Supplemental Figure 3. Factor loadings of the 4 multimorbidity patterns for each condition in CHARLS 2018

Supplemental Figure 4. Average factor scores for the four multimorbidity trajectory groups, based on sensitivity analysis using participants with complete data for at least 3 waves

Supplemental Figure 5. Average factor scores for the four multimorbidity trajectory groups, based on sensitivity analyses that only excluded participants with multimorbidity at baseline

Supplemental Table 1. Baseline characteristic of the analytical sample from the CHARLS study

| Characteristic | No multimorbidity (N=3141) | New-onset multimorbidity (N=2407) | P Value |
| --- | --- | --- | --- |
| Sex, n (%)^a^ |  |  | 0.023 |
| Male | 1629 (51.9) | 1174 (48.8) |  |
| Female | 1512 (48.1) | 1233 (51.2) |  |
| Age(years),mean±SD^b^ | 56.4±8.7 | 57.7±8.5 | <.001 |
| Self-rated health, n (%)^a^ |  |  | <.001 |
| Poor | 218 (9.7) | 322 (18.4) |  |
| Fair | 996 (44.3) | 930 (53.2) |  |
| Good | 1032 (46.0) | 497 (28.4) |  |
| Education, n (%)^a^ |  |  | <.001 |
| No formal education | 1254 (39.9) | 1101 (45.7) |  |
| Primary school | 687 (21.9) | 523 (21.7) |  |
| Middle school or above | 1200 (38.2) | 783 (32.6) |  |
| BMI (kg/m^2^), n (%) ^a^ |  |  | <.001 |
| <18.5 | 152 (5.8) | 116 (5.8) |  |
| 18.5-22.9 | 1272 (48.7) | 853 (42.9) |  |
| 23.0-27.4 | 938 (35.8) | 770 (38.7) |  |
| ≧27.5 | 255 (9.7) | 250 (12.6) |  |
| Occupation, n (%)^a^ |  |  | <.001 |
| Agricultural work | 1657 (53.0) | 1288 (53.7) |  |
| Non-agricultural work | 870 (27.8) | 531 (22.1) |  |
| Unemployed/retired | 601 (19.2) | 581 (24.2) |  |
| Number of chronic conditions, mean±SD^b^ | 0.3±0.5 | 0.7±0.5 | <.001 |
| ADL disability, n (%)^a^ |  |  | <.001 |
| No | 2905 (93.6) | 2067 (86.7) |  |
| Yes | 200 (6.4) | 316 (13.3) |  |
| Smoking, n (%)^a^ |  |  | 0.642 |
| No | 1846 (58.8) | 1430 (59.4) |  |
| Yes | 1294 (41.2) | 977 (40.6) |  |
| Alcohol drinking, n (%)^a^ |  |  | 0.627 |
| No | 1862 (59.3) | 1442 (60.0) |  |
| Yes | 1276 (40.7) | 962 (40.0) |  |
| Sleep duration(h/day), n (%)^a^ |  |  | <.001 |
| <7 | 1242 (42.2) | 1116 (49.6) |  |
| 7-8 | 1443 (48.9) | 928 (41.2) |  |
| >8 | 263 (8.9) | 207 (9.2) |  |
| Marital status, n (%)^a^ |  |  | 0.197 |
| Married/cohabitation | 2871 (91.4) | 2176 (90.4) |  |
| Single | 270 (8.6) | 231 (9.6) |  |
| Annual household expenditure(yuan), n (%)^a^ |  |  | 0.358 |
| ≤2800 | 712 (26.7) | 590 (28.3) |  |
| 2801-4846 | 726 (27.2) | 525 (25.2) |  |
| 4847-8325 | 647 (24.3) | 520 (25.0) |  |
| >8325 | 582 (21.8) | 448 (21.5) |  |
| Social engagement, n (%)^a,c^ |  |  | 0.547 |
| No | 1571 (53.0) | 1223 (53.9) |  |
| Yes | 1391 (47.0) | 1047 (46.1) |  |
| Location of residence, n (%)^a^ |  |  | 0.151 |
| Rural | 2082 (66.3) | 1551 (64.4) |  |
| Urban | 1059 (33.7) | 856 (35.6) |  |
| Health insurance, n (%)^a,d^ |  |  | 0.327 |
| No | 229 (7.3) | 159 (6.6) |  |
| Yes | 2905 (92.7) | 2240 (93.4) |  |

Notes:

^a^ Pearson chi-square tests for categorical variables.

^b^ One-way analysis of variance tests for continuous variables.

^c^ Social engagement (including Interacted with friend; Played Ma-jong, chess, cards, or went to a community club; Went to a sporting event, participated in a social group, or participated in some other sort of club; Took part in a community-related organization; Took part in voluntary or charity work ;or attended an educational or training course) was dichotomized: “yes: the respondent does participate in one of the social activities ”vs “no: never”.

^d^ Health insurance was categorized as “no: not covered by any public health insurance plan,” or “yes: covered by at least one type of public health insurance plan.”

Supplemental Table 2. Comparison of baseline characteristics between participants included (n = 5,548) and excluded (n =12,160)

| Characteristic | Excluded (N=12160) | Included (N=5548) | P Value* |
| --- | --- | --- | --- |
| Sex, n (%)^a^ |  |  | <.001 |
| Male | 5673 (46.7) | 2803 (50.5) |  |
| Female | 6485 (53.3) | 2745 (49.5) |  |
| Age(years),mean±SD^b^ | 59.5±10.7 | 57.0±8.6 | <.001 |
| Self-rated health, n (%)^a^ |  |  | <.001 |
| Poor | 2629 (30.5) | 540 (13.5) |  |
| Fair | 3992 (46.3) | 1926 (48.2) |  |
| Good | 1997 (23.2) | 1529 (38.3) |  |
| Education, n (%)^a^ |  |  | <.001 |
| No formal education | 5594 (46.1) | 2355 (42.5) |  |
| Primary school | 2612 (21.5) | 1210 (21.8) |  |
| Middle school or above | 3937 (32.4) | 1983 (35.7) |  |
| BMI(kg/m^2^), n (%) ^a^ |  |  | <.001 |
| <18.5 | 679 (7.5) | 268 (5.8) |  |
| 18.5-22.9 | 3592 (39.9) | 2125 (46.1) |  |
| 23.0-27.4 | 3392 (37.6) | 1708 (37.1) |  |
| ≥27.5 | 1348 (15.0) | 505 (11.0) |  |
| Occupation, n (%)^a^ |  |  | <.001 |
| Agricultural work | 5016 (42.1) | 2945 (53.3) |  |
| Non-agricultural work | 2448 (20.5) | 1401 (25.3) |  |
| Unemployed/retired | 4462 (37.4) | 1182 (21.4) |  |
| Number of chronic conditions,mean±SD^b^ | 1.8±1.5 | 0.5±0.5 | <.001 |
| ADL disability, n (%)^a^ |  |  | <.001 |
| No | 9506 (80.1) | 4972 (90.6) |  |
| Yes | 2368 (19.9) | 516 (9.4) |  |
| Smoking, n (%)^a^ |  |  | 0.007 |
| No | 7352 (61.2) | 3276 (59.1) |  |
| Yes | 4660 (38.8) | 2271 (40.9) |  |
| Alcohol drinking, n (%)^a^ |  |  | 0.006 |
| No | 7414 (61.8) | 3304 (59.6) |  |
| Yes | 4585 (38.2) | 2238 (40.4) |  |
| Sleep duration(h/day), n (%)^a^ |  |  | <.001 |
| <7 | 5747 (52.8) | 2358 (45.4) |  |
| 7 -8 | 4242 (38.9) | 2371 (45.6) |  |
| >8 | 903 (8.3) | 470 (9.0) |  |
| Marital status, n (%)^a^ |  |  | <.001 |
| Married/cohabitation | 10370 (85.5) | 5047 (91.0) |  |
| Single | 1757 (14.5) | 501 (9.0) |  |
| Household expenditure(yuan), n (%)^a^ |  |  | <.001 |
| ≤2800 | 2391 (23.9) | 1302 (27.4) |  |
| 2801-4846 | 2445 (24.4) | 1251 (26.3) |  |
| 4847-8325 | 2521 (25.1) | 1167 (24.6) |  |
| >8325 | 2662 (26.6) | 1030 (21.7) |  |
| Social engagement, n (%)^a,c^ |  |  | 0.721 |
| No | 5837 (53.1) | 2794 (53.4) |  |
| Yes | 5155 (46.9) | 2438 (46.6) |  |
| Location of residence, n (%)^a^ |  |  | <.001 |
| Rural | 6904 (56.8) | 3633 (65.5) |  |
| Urban | 5256 (43.2) | 1915 (34.5) |  |
| Health insurance, n (%)^a,d^ |  |  | 0.002 |
| No | 997 (8.4) | 388 (7.0) |  |
| Yes | 10937 (91.6) | 5145 (93.0) |  |

Notes:

^a^ Pearson chi-square tests for categorical variables.

^b^ One-way analysis of variance tests for continuous variables.

^c^ Social engagement (including Interacted with friend; Played Ma-jong, chess, cards, or went to a community club; Went to a sporting event, participated in a social group, or participated in some other sort of club; Took part in a community-related organization; Took part in voluntary or charity work ;or attended an educational or training course) was dichotomized: “yes: the respondent does participate in one of the social activities ”vs “no: never”.

^d^ Health insurance was categorized as “no: not covered by any public health insurance plan,” or “yes: covered by at least one type of public health insurance plan.”

Supplemental Table 3. Association of multimorbidity and incident disability based on the GEE model among participants excluded from the current analysis

|  | Model 1^a^ | Model 2^b^ | Model 3^c^ |
| --- | --- | --- | --- |
| Multimorbidity trajectory | OR (95% CI) | OR (95% CI) | OR (95% CI) |
| No multimorbidity (referent) | 1.00 | 1.00 | 1.00 |
| multimorbidity | 2.23***(2.03 - 2.45) | 2.25***(1.98 - 2.55) | 1.64***(1.41 - 1.90) |

Notes:

Abbreviations: OR, odds ratio; CI, confidence intervals.

a. Adjusted for time.

b. Adjusted for time and sociodemographic characteristics variables, including age, gender, marital status, living area, occupation, annual household expenditure, social participation, and public health insurance status.

c. Adjusted for time, sociodemographic characteristics variables, and health-related characteristics (self-rated health, BMI, smoking, alcohol drinking, and sleep duration).

*** p<.001, ** p<.01, * p<.05.

Supplemental Table 4. Association of multimorbidity trajectories and incident disability in the analysis that only excluded participants with multimorbidity at baseline

|  | Model 1^a^ | Model 2^b^ | Model 3^c^ |
| --- | --- | --- | --- |
| Multimorbidity trajectory | OR (95% CI) | OR (95% CI) | OR (95% CI) |
| No multimorbidity (referent) | 1.00 | 1.00 | 1.00 |
| New-onset multimorbidity | 2.22***(1.99 - 2.47) | 2.30***(2.01 - 2.63) | 1.82***(1.56 - 2.12) |
| Multimorbidity trajectory groups  (ref = no multimorbidity) |  |  |  |
| Cardiometabolic | 2.27***(1.98 - 2.60) | 2.49***(2.11 - 2.93) | 2.07***(1.72 - 2.49) |
| Digestive-arthritic | 1.84***(1.57 - 2.17) | 2.08***(1.70 - 2.53) | 1.55***(1.24 - 1.94) |
| Cardiometabolic/Brain | 2.55***(2.17 - 3.00) | 2.38***(1.94 - 2.92) | 1.87***(1.48 - 2.36) |
| Respiratory | 2.30***(1.81 - 2.93) | 1.85***(1.33 - 2.57) | 1.36(0.92 - 2.01) |

Notes:

Abbreviations: OR, odds ratio; CI, confidence intervals.

a. Adjusted for time.

b. Adjusted for time and sociodemographic characteristics variables, including age, gender, marital status, living area, occupation, annual household expenditure, social participation, and public health insurance status.

c. Adjusted for time, sociodemographic characteristics variables, and health-related characteristics (self-rated health, BMI, smoking, alcohol drinking, and sleep duration).

*** p<.001, ** p<.01, * p<.05.

Supplemental Table 5. The impact of single morbidity on the association between multimorbidity trajectories and incident disability

|  | Model 1^a^ | Model 2^b^ | Model 3^c^ |
| --- | --- | --- | --- |
| Multimorbidity trajectory | OR (95% CI) | OR (95% CI) | OR (95% CI) |
| No morbidity (referent) | 1.00 | 1.00 | 1.00 |
| single morbidity | 1.55***(1.28 - 1.88) | 1.44**(1.13 - 1.84) | 1.33*(1.02 - 1.73) |
| Multimorbidity trajectory groups  (ref = no morbidity) |  |  |  |
| Cardiometabolic | 2.54***(2.04 - 3.17) | 2.80***(2.14 - 3.67) | 2.30***(1.71 - 3.09) |
| Digestive-arthritic | 2.68***(2.14 - 3.35) | 2.79***(2.13 - 3.67) | 1.99***(1.47 - 2.70) |
| Cardiometabolic/Brain | 3.30***(2.63 - 4.15) | 2.92***(2.19 - 3.90) | 2.41***(1.76 - 3.30) |
| Respiratory | 2.62***(1.91 - 3.57) | 2.39***(1.59 - 3.59) | 1.69*(1.06 - 2.69) |

Notes:

Abbreviations: OR, odds ratio; CI, confidence intervals.

a. Adjusted for time.

b. Adjusted for time and sociodemographic characteristics variables, including age, gender, marital status, living area, occupation, annual household expenditure, social participation, and public health insurance status.

c. Adjusted for time, sociodemographic characteristics variables, and health-related characteristics (self-rated health, BMI, smoking, alcohol drinking, and sleep duration).

*** p<.001, ** p<.01, * p<.05.

Supplemental Table 6. The goodness of fit for the EFA models

|  | | CFI | TLI | RMSEA | SRMR |
| --- | --- | --- | --- | --- | --- |
| Two-factors | 0.895 | | 0.845 | 0.032 | 0.086 |
| Three-factors | 0.974 | | 0.952 | 0.018 | 0.062 |
| Four-factors | 0.986 | | 0.966 | 0.015 | 0.043 |
| Five-factors | 0.995 | | 0.982 | 0.011 | 0.033 |

Notes: CFI: Comparative fit index; TLI: Tucker and Lewis index; RMSEA: Root mean squared error of approximation; SRMR :Standardized Root Mean Square Residual; The cutoff values for good model fits were adopted as CFI, TLI > 0.95; SRMR, RMSEA < 0.06.

Supplemental Table 7. Model fitting statistics for participants with new onset of multimorbidity

| Fit statistic | Number of classes | | | | | |
| --- | --- | --- | --- | --- | --- | --- |
|  | 1 | 2 | 3 | 4 | 5 | 6 |
| BIC* | -11489.44 | -8408.67 | -7067.33 | -5017.42 | -4282.39 | -3638.58 |
| AIC* | -11443.15 | -8324.77 | -6966.07 | -4869.87 | -4085.66 | -3404.24 |
| Class proportion^¶^ | Class1,100% | Class1,57.38% | Class1,61.55% | Class1,33.84% | Class1,9.07% | Class1,3.75% |
|  |  | Class2,42.62% | Class2,28.87% | Class2,31.61% | Class2,41.74% | Class2,32.80% |
|  |  |  | Class3,9.58% | Class3,25.60% | Class3,35.97% | Class3,27.62% |
|  |  |  |  | Class4,8.95% | Class4,6.82% | Class4,23.55% |
|  |  |  |  |  | Class5,6.40% | Class5,6.49% |
|  |  |  |  |  |  | Class6,5.79% |
| AvePP^‡^ |  | Class1,0.96 | Class1,0.96 | Class1,0.90 | Class1,0.95 | Class1,0.99 |
|  |  | Class2,0.96 | Class2,0.94 | Class2,0.94 | Class2,0.95 | Class2,0.90 |
|  |  |  | Class3,0.98 | Class3,0.93 | Class3,0.94 | Class3,0.91 |
|  |  |  |  | Class4,0.98 | Class4,0.99 | Class4,0.94 |
|  |  |  |  |  | Class5,0.92 | Class5,0.97 |
|  |  |  |  |  |  | Class6,0.92 |

Notes: AIC: Akaike’s information criterion;BIC: Bayesian information criteria; AvePP: average posterior probabilities;

*A lower absolute value suggests a better model fit ;¶No less than 5% of total count in a class ;‡A higher value is better (preferably > 0.7 in a class).

Supplemental Table 8. The final four-group trajectory model of multimorbidity pattern for participants with new onset of multimorbidity

Cardio-metabolic

| Trajectory group | Parameter | Maximum likelihood estimates | | | |
| --- | --- | --- | --- | --- | --- |
|  |  | Est. | SE | Z value | p value |
| Cardiometabolic(N=821, 34.11%) | Intercept | 0.177 | 0.035 | 4.985 | 0.000 |
|  | Linear | -0.196 | 0.034 | -5.697 | 0.000 |
|  | Quadratic | 0.081 | 0.007 | 11.291 | 0.000 |
| Digestive-arthritic(N=753, 31.28%) | Intercept | -0.075 | 0.016 | -4.670 | 0.000 |
|  | Linear | 0.085 | 0.006 | 13.851 | 0.000 |
| Cardiometabolic/Brain(N=618, 25.68%) | Intercept | 0.593 | 0.117 | 5.067 | 0.000 |
|  | Linear | -0.468 | 0.180 | -2.608 | 0.009 |
|  | Quadratic | 0.319 | 0.080 | 4.005 | 0.000 |
|  | Cubic | -0.042 | 0.011 | -3.925 | 0.000 |
| Respiratory(N=215, 8.93%) | Intercept | -0.135 | 0.029 | -4.653 | 0.000 |
|  | Linear | 0.132 | 0.011 | 12.206 | 0.000 |

Brain

| Trajectory group | Parameter | Maximum likelihood estimates | | | |
| --- | --- | --- | --- | --- | --- |
|  |  | Est. | SE | Z value | p value |
| Cardiometabolic(N=821, 34.11%) | Intercept | 0.110 | 0.020 | 5.543 | 0.000 |
|  | Linear | -0.103 | 0.019 | -5.438 | 0.000 |
|  | Quadratic | 0.038 | 0.004 | 9.677 | 0.000 |
| Digestive-arthritic(N=753, 31.28%) | Intercept | -0.075 | 0.009 | -8.127 | 0.000 |
|  | Linear | 0.015 | 0.003 | 4.573 | 0.000 |
| Cardiometabolic/Brain(N=618, 25.68%) | Intercept | 0.054 | 0.011 | 4.999 | 0.000 |
|  | Linear | 0.098 | 0.004 | 24.048 | 0.000 |
| Respiratory(N=215, 8.93%) | Intercept | 0.124 | 0.007 | 17.908 | 0.000 |

Digestive-arthritic

| Trajectory group | Parameter | Maximum likelihood estimates | | | |
| --- | --- | --- | --- | --- | --- |
|  |  | Est. | SE | Z value | p value |
| Cardiometabolic(N=821, 34.11%) | Intercept | 0.609 | 0.097 | 6.277 | 0.000 |
|  | Linear | -0.783 | 0.150 | -5.224 | 0.000 |
|  | Quadratic | 0.402 | 0.067 | 5.991 | 0.000 |
|  | Cubic | -0.053 | 0.009 | -5.927 | 0.000 |
| Digestive-arthritic(N=753, 31.28%) | Intercept | 1.280 | 0.099 | 12.922 | 0.000 |
|  | Linear | -1.551 | 0.152 | -10.177 | 0.000 |
|  | Quadratic | 0.838 | 0.068 | 12.355 | 0.000 |
|  | Cubic | -0.119 | 0.009 | -13.107 | 0.000 |
| Cardiometabolic/Brain(N=618, 25.68%) | Intercept | 0.357 | 0.109 | 3.272 | 0.001 |
|  | Linear | -0.577 | 0.168 | -3.443 | 0.001 |
|  | Quadratic | 0.310 | 0.074 | 4.164 | 0.000 |
|  | Cubic | -0.041 | 0.010 | -4.173 | 0.000 |
| Respiratory(N=215, 8.93%) | Intercept | 0.656 | 0.183 | 3.587 | 0.000 |
|  | Linear | -0.915 | 0.280 | -3.267 | 0.001 |
|  | Quadratic | 0.485 | 0.124 | 3.917 | 0.000 |
|  | Cubic | -0.066 | 0.016 | -4.000 | 0.000 |

Respiratory

| Trajectory group | Parameter | Maximum likelihood estimates | | | |
| --- | --- | --- | --- | --- | --- |
|  |  | Est. | SE | Z value | p value |
| Cardiometabolic(N=821, 34.11%) | Intercept | 0.034 | 0.004 | 9.043 | 0.000 |
| Digestive-arthritic(N=753, 31.28%) | Intercept | -0.036 | 0.009 | -3.972 | 0.000 |
|  | Linear | 0.030 | 0.003 | 8.916 | 0.000 |
| Cardiometabolic/Brain(N=618, 25.68%) | Intercept | -0.028 | 0.010 | -2.796 | 0.005 |
|  | Linear | 0.022 | 0.004 | 5.845 | 0.000 |
| Respiratory(N=215, 8.93%) | Intercept | 0.868 | 0.115 | 7.541 | 0.000 |
|  | Linear | -1.020 | 0.176 | -5.780 | 0.000 |
|  | Quadratic | 0.555 | 0.078 | 7.094 | 0.000 |
|  | Cubic | -0.072 | 0.010 | -6.875 | 0.000 |

Note: Est.: parameter estimate,SE: standard error of parameter estimate


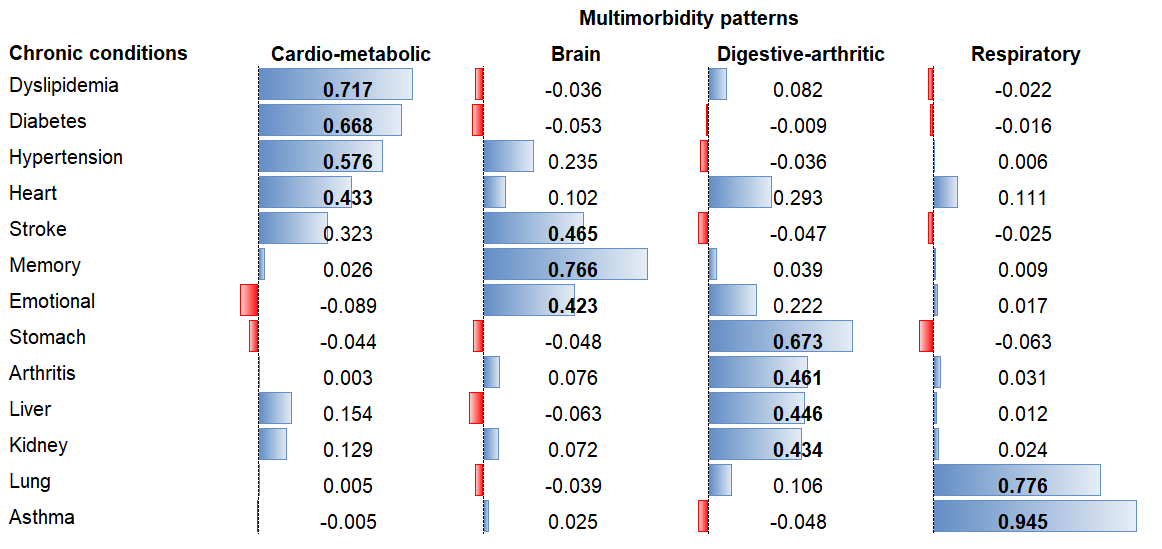


Supplemental Figure 1 . Factor loadings of the 4 multimorbidity patterns for each condition in CHARLS 2013


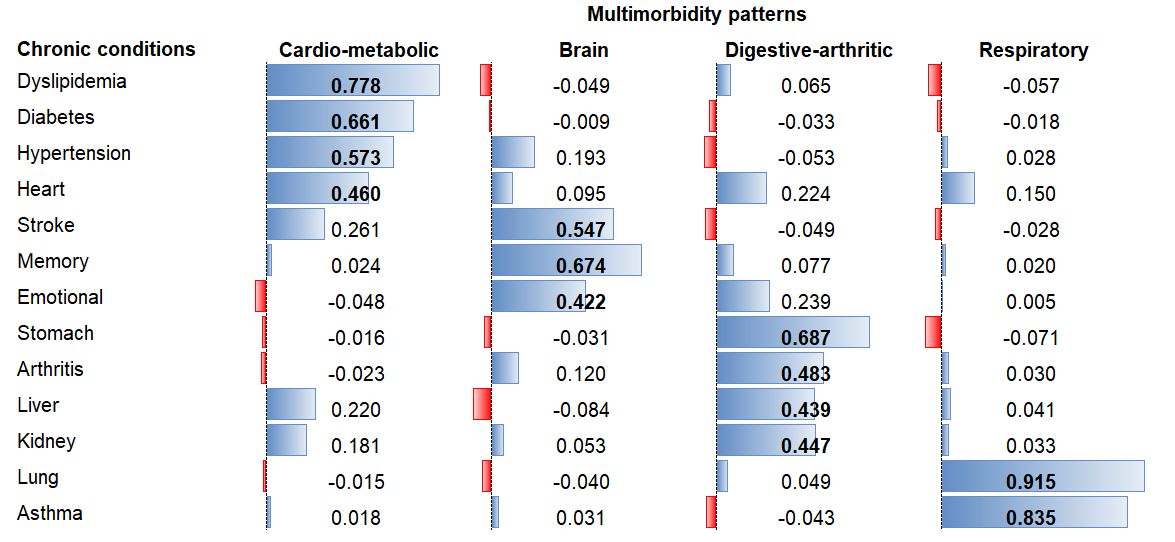


Supplemental Figure 2. Factor loadings of the 4 multimorbidity patterns for each condition in CHARLS 2015


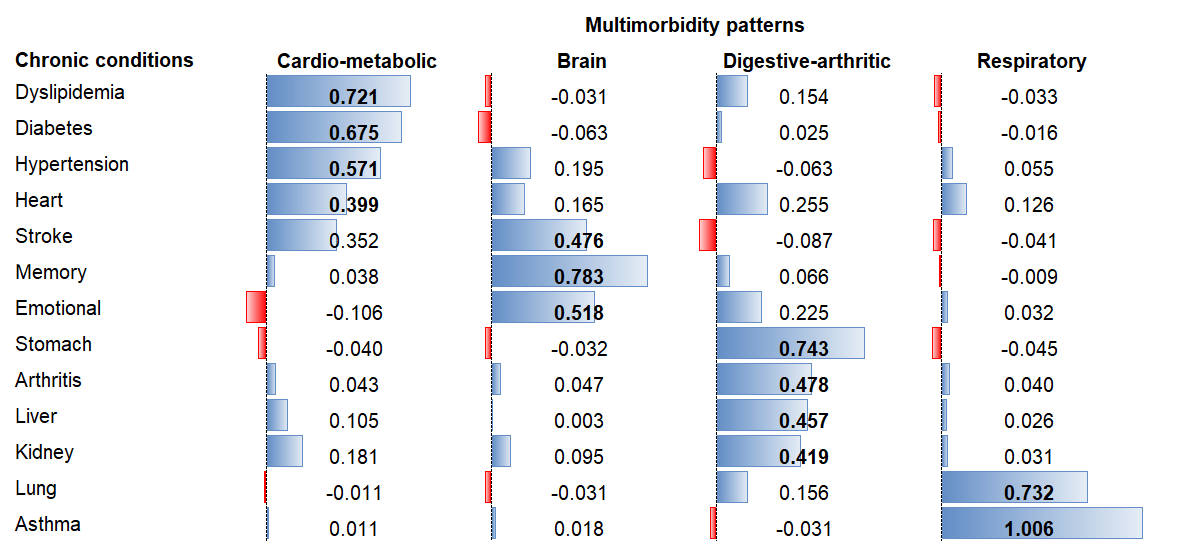


Supplemental Figure 3. Factor loadings of the 4 multimorbidity patterns for each condition in CHARLS 2018


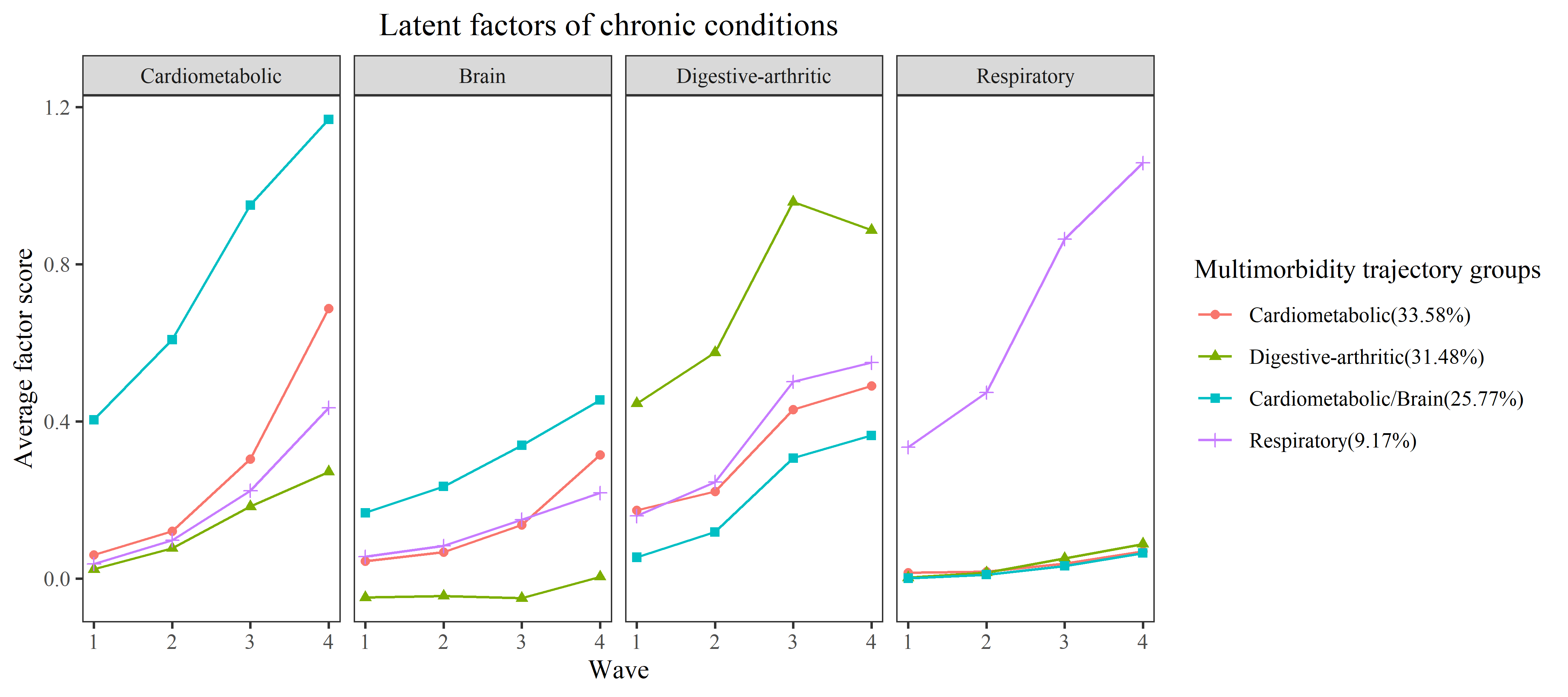


Supplemental Figure 4. Average factor scores for the four multimorbidity trajectory groups, based on sensitivity analyses using participants with complete data for at least 3 waves


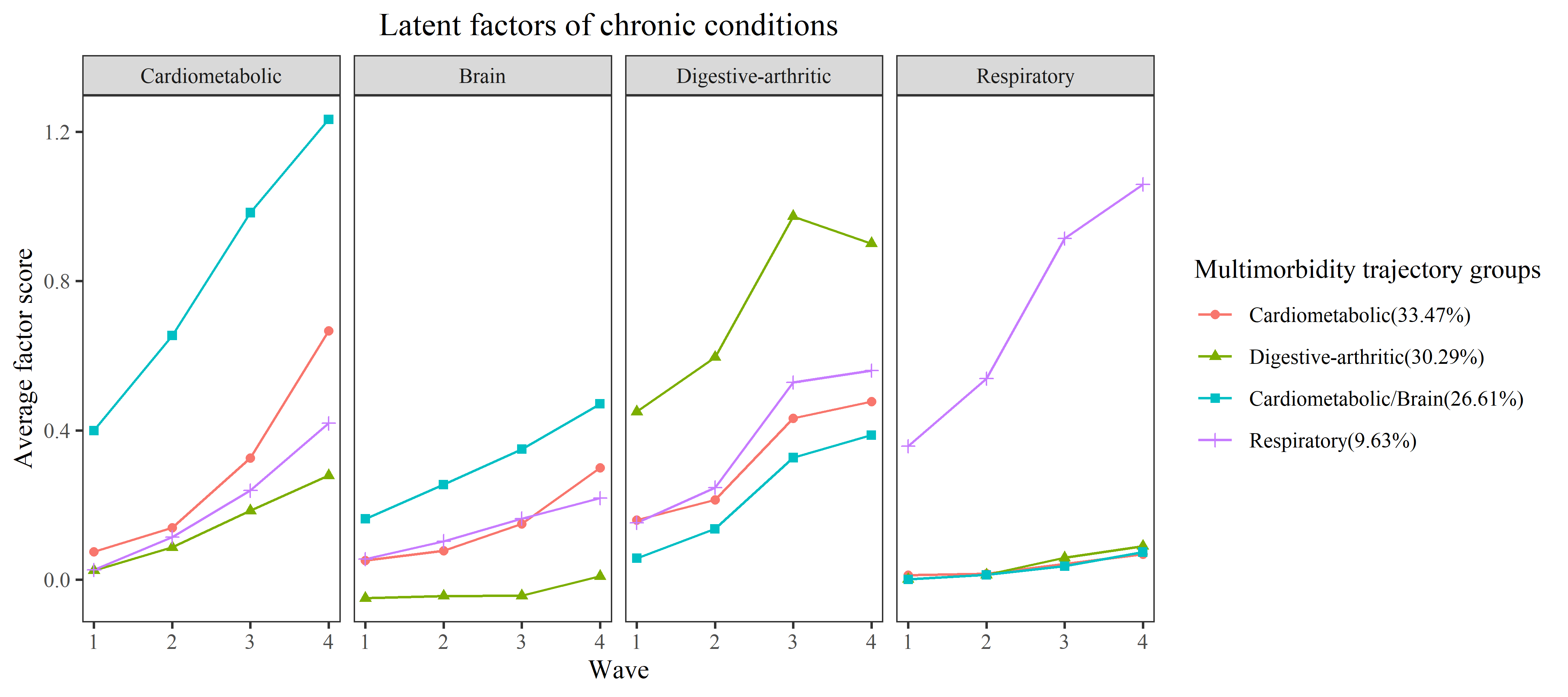


Supplemental Figure 5. Average factor scores for the four multimorbidity trajectory groups, based on sensitivity analyses that only excluded participants with multimorbidity at baseline
